# PCR-confirmed severe geographic HSV-1 keratitis associated with systemic JAK inhibitor therapy

**DOI:** 10.1186/s12348-026-00601-1

**Published:** 2026-05-23

**Authors:** Dimitri Roels, Elke O. Kreps, Isabeau Houben, Anke Delie, Hilde Lapeere, Bart P. Leroy, Ilse Claerhout, Tessa Kerre

**Affiliations:** 1 Department of Ophthalmology, Ghent University Hospital, Corneel Heymanslaan 10, Ghent, 9000 Belgium; 2 Department of Hematology, Ghent University Hospital, Ghent, Belgium; 3 Department of Dermatology, Ghent University Hospital, Ghent, Belgium; 4 Department of Head & Skin, Ghent University, Ghent, Belgium; 5 Center for Medical Genetics, Ghent University Hospital, Ghent, Belgium; 6 Department of Ophthalmology, AZ Maria Middelares Hospital, Ghent, Belgium; 7 Department of Internal Medicine and Pediatrics, Ghent University, Ghent, Belgium

**Keywords:** Janus kinase inhibitors, Baricitinib, Upadacitinib, Ruxolitinib, Abrocitinib, Herpes simplex, Herpetic keratitis

## Abstract

**Background:**

Janus kinase inhibitors (JAKi) are successfully used for the treatment of refractory systemic autoimmune diseases. However, JAK-inhibition is known to increase the risk of infection. Herpes Zoster reactivation is the most recognised infectious complication of JAKi. Events involving herpes simplex are reported less frequently than zoster and have generally received less attention. Our goal is to raise awareness of JAKi-associated herpes simplex keratitis.

**Methods:**

This paper describes four cases of severe herpes simplex keratitis in patients treated with oral JAK inhibitors.

**Results:**

All patients presented with a geographic epithelial keratitis. One patient presented with bilateral involvement. Viral PCR of corneal swabs demonstrated the presence of HSV-1 and absence of VZV in all patients, confirming the diagnosis of herpes simplex keratitis. The patients were treated with various oral JAK inhibitors (baricitinib, upadacitinib, ruxolitinib and abrocitinib), which demonstrated an underlying class effect. Finally, the time lapse also supports an association between the use of oral JAKi and herpetic keratitis, as evidenced by the development of HSV1-related keratitis shortly after the start of oral JAKi in 3 of the 4 patients.

**Conclusions:**

Clinicians should be aware of the association between the use of oral JAKi and herpes simplex keratitis. Not only can HSV1-related keratitis manifest itself in this patient group as a severe geographic keratitis, it can also occur bilaterally. An urgent ophthalmological evaluation is recommended for patients taking oral JAKi who present with unilateral or bilateral red, painful eye(s), even in the absence of periocular signs of Herpes Zoster infection.

## Summary box

**What was known before**

- Janus kinase inhibitors (JAKi) are successfully used for the treatment of refractory systemic autoimmune diseases, myeloproliferative diseases, and GVHD.
- JAK-inhibition increases the risk of infection.
- Herpes Zoster reactivation is the most frequently recognised infectious complication of JAK inhibitors.

**What this study adds**

- Clinicians need to be aware of the association between oral JAKi use and herpes simplex keratitis.
- Not only can HSV1-related keratitis manifest itself in this patient group as a severe geographic keratitis, it can also occur bilaterally.
- Oral antiviral prophylaxis may be considered for high-risk patients taking JAKi, such as those with a history of HSV keratitis, severe atopic dermatitis, high-dose JAKi therapy, or concomitant steroid use.

## Introduction

The JAK pathway is recognised as a key player in the immune dysregulation in many immune mediated inflammatory diseases (IMIDs). Janus kinase inhibitors (JAKi) are targeted disease-modifying small molecule agents used for the treatment of refractory autoimmune diseases such as atopic dermatitis (AD), rheumatoid arthritis (RA), spondyloarthritis (SpA), and ulcerative colitis (UC) [1–3]. The JAK inhibitor tofacitinib (non-selective) was licensed in 2012, followed by baricitinib (JAK1&2 selective) in 2017, upadacitinib (JAK1 selective) in 2019, filgotinib (JAK1 selective) in 2020 and abrocitinib (JAK1 selective) in 2022, by the European Medical Agency (EMA) [4]. Ruxolitinib (JAK1&2 selective) is used in the treatment of patients with myelofibrosis, polycythaemia rubra vera and refractory graft-versus-host disease after allogeneic stem cell transplantation [4]. Despite the greater efficacy of these oral JAKi over other systemic treatments demonstrated in clinical trials, clinicians remain conservative in prescribing them because of safety concerns surrounding JAKi [1, 2]. Frequently reported adverse events in clinical trials investigating oral JAKi for AD included both mild, transient side effects, such as headache and nausea, and more concerning complications, including serious infections, opportunistic infections, herpes zoster, herpes simplex, nasopharyngitis, acne, anemia, neutropenia, thrombocytopenia, elevated creatine phosphokinase levels, and elevated cholesterol levels [1, 5]. Tsai et al. [1] showed in a population-based cohort study that patients receiving oral JAKi for atopic dermatitis had significantly higher risks of systemic (non-ocular) herpes infection than patients receiving dupilumab (herpes simplex, HR 1.64, 95% CI 1.03–2.61; herpes zoster, HR 2.51, 95% CI 1.14–5.52). However, the causal link between oral JAKi and herpetic keratitis is uncertain [3, 6]. In a dose escalation study of tofacitinib in refractory dermatomyositis by Ida et al. [7], one patient discontinued tofacitinib 16 months after dose escalation because of herpes simplex keratitis.

This study describes four consecutive cases of severe herpes simplex keratitis in patients treated with oral JAK inhibitors. All patients were referred to the tertiary ophthalmology department at Ghent University Hospital (Belgium). To the best of our knowledge, there have been no detailed case descriptions of herpetic keratitis in patients receiving JAKi to date. Our goal is to raise awareness of this serious complication by describing the morphological characteristics and outcomes of JAKi-associated herpetic keratitis.

## Case series

### Case 1

**Fig. 1:**
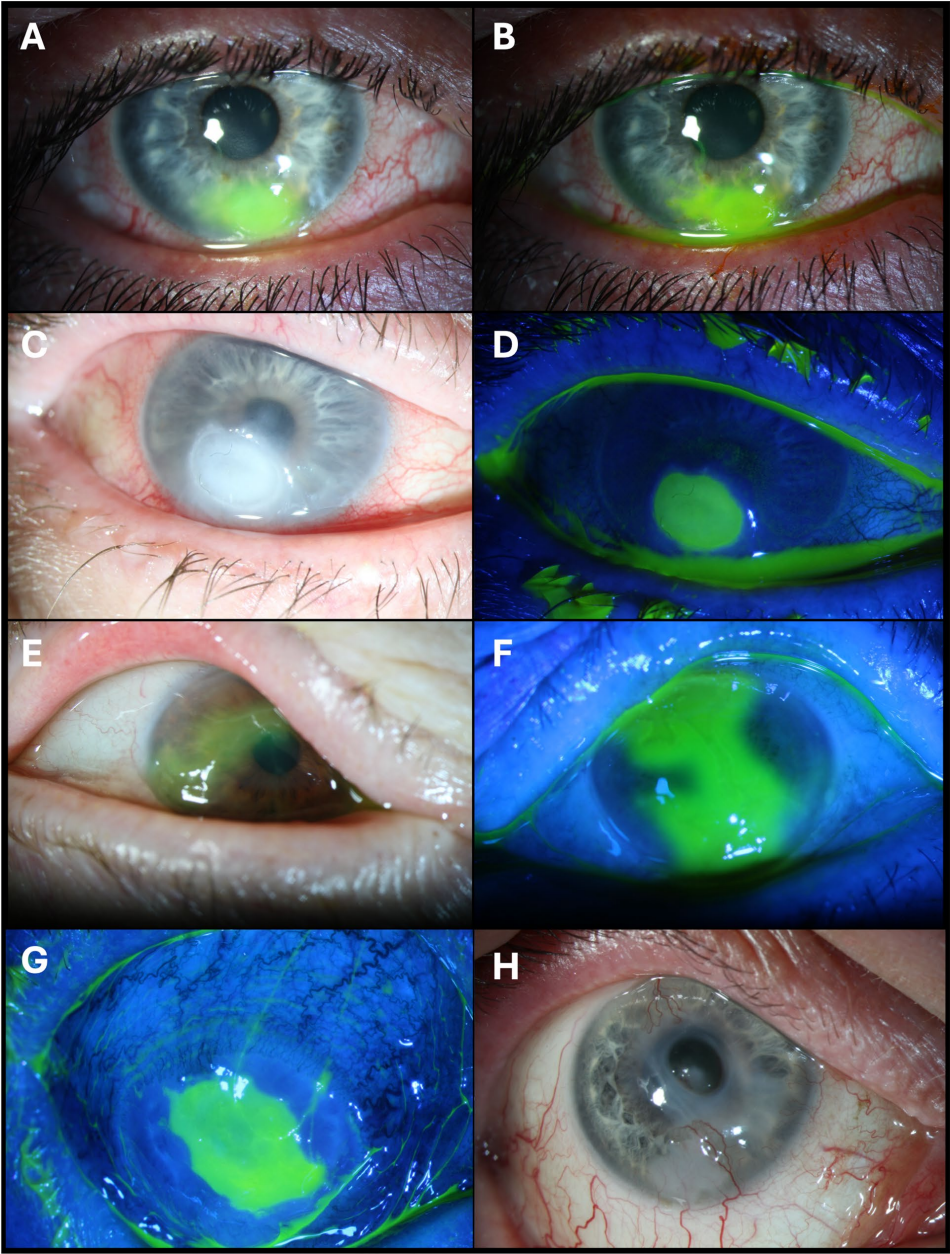
Anterior segment photography illustrating herpetic geographic epithelial keratitis at presentation in Case 1 (**A** & **B**), after 2 weeks of topical and oral antivirals in Case 2 (**C** & **D**), at presentation in Case 3 in the right (**E**) and left eye (**F**), and after 3 weeks of topical dexamethasone and oral antivirals in Case 4 (**G**). Notice progression towards a prepupillary descemetocele, accompanied by pronounced corneal neovascularization in Case 4 (**H**). Fluorescein was used to stain the epithelial defect. Pictures **D**, **F** and **G** were taken using a cobalt blue filter

A 36-year-old woman was referred by the dermatology department due to eczema herpeticum with newly developed vision loss, irritation, and redness in her right eye. She was known to have refractory atopic dermatitis, for which baricitinib once daily had been started four months earlier. As baricitinib was initiated as part of a blinded study, the dosage was either 2 mg or 4 mg per day. At the time of referral, she was not taking any other immunosuppressants. She received maintenance treatment for chronic atopic conjunctivitis with preservative-free artificial tears (PFAT), fluorometholone 0.1%, and cyclosporine 0.1% eye drops. She had no history of contact lens use, trauma, eye surgery, or herpetic eye disease. The patient had received a VZV vaccination six months prior. In the context of eczema herpeticum, the dermatology department had already started her on oral valacyclovir at a dose of three grams per day. Her best-corrected visual acuity (BCVA) was 20/25. Slit lamp exam showed ciliary injection, a geographic corneal epithelial defect inferiorly with dendritic extensions toward the pupil, and 1 + anterior chamber cells without endothelial plaque or hypopyon (Fig. 1A & B). Intraocular pressure (IOP) was 13 mmHg, and the fundus examination was normal. The left eye showed no abnormalities. A corneal swab for viral PCR confirmed the presence of herpes simplex virus type 1 (HSV-1) and the absence of varicella zoster virus (VZV). Following consultation with the dermatology department, baricitinib was discontinued. Treatment consisted of 0.15% ganciclovir ointment five times daily, fluorometholone 0.1% once daily, 1% cyclopentolate twice daily, and intensive PFAT. Oral valacyclovir was maintained at a dose of three grams per day for three weeks, after which the antiviral therapy was gradually tapered and finally stopped after six months. After three weeks, the epithelial defect had closed, and there was no more anterior chamber inflammation. The final BCVA was 20/25 (Table 1).

### Case 2

A 54-year-old man was referred by his own ophthalmologist for an evaluation of a painful, red eye and blurred vision which had been present for two weeks. His ophthalmologist had noticed a large geographic corneal epithelial defect in the patient’s right eye and prescribed a combination of topical ganciclovir 0.15% ointment three times a day and oral acyclovir at a dose of 4 g per day. Upon presentation at our hospital, BCVA was 20/40. Slit lamp exam revealed a round corneal epithelial defect with whitish infiltration and raised edges, 1 + cells in the anterior chamber, and scattered endothelial precipitates without hypopyon (Fig. 1C & D). IOP was 12 mmHg, and the fundus examination was normal. The left eye showed the characteristic features of chronic atopic blepharoconjunctivitis with no other abnormalities. There was no history of contact lens use, trauma, or herpetic eye disease. There was no history of ocular laser treatment or surgery, except for strabismus surgery in childhood. The patient had been treated for three years for severe, refractory atopic dermatitis with oral upadacitinib once daily. As upadacitinib was initiated as part of a blinded study, the dosage was either 15 mg or 30 mg per day. At the time of referral, he was also being treated with mometasone 0.1% cream twice daily. He was not taking any other immunosuppressants. Corneal swabs were taken for culture and viral RealTime PCR. The results confirmed the presence of both HSV-1 (Ct value 27.73) and *Staphylococcus aureus*, and the absence of VZV. Following consultation with the dermatology department, upadacitinib was discontinued. Topical treatment was changed to hourly alternating cefazolin 5% and tobramycin 1.4% in combination with dexamethasone 0.1% once daily. Oral acyclovir was switched to valacyclovir 3 g per day, and doxycycline 100 mg twice daily was added. Despite intensive treatment, BCVA decreased to counting fingers (< 20/200) and neurotrophic keratopathy developed. After seven months, the epithelial defect closed using a combination of intensive PFAT, autologous serum eye drops, and ultimately a total amniotic membrane transplant. However, BCVA remained limited to counting fingers. One year later, cataract surgery improved his final BCVA to 20/63. Oral valacyclovir was tapered to 500 mg/day and continued as prophylactic treatment (Table 1).

### Case 3

A 78-year-old man was referred by his general practitioner (GP) due to photophobia, epiphora, and a foreign body sensation in both eyes (synchronous) for the past two weeks. His GP initially prescribed a combination of tobramycin and dexamethasone eye drops but discontinued them after one week due to lack of improvement. Subsequently, topical antibiotics were replaced by PFAT. Upon presentation, BCVA was 20/400 in the right eye and 20/200 in the left eye. Slit lamp exam revealed an elongated corneal erosion extending from the temporal side of the cornea, along the superior pupil margin to the superonasal side of the cornea in the right eye, as well as a large geographic corneal epithelial defect extending from the inferior limbus to the superior limbus with dendritic extensions in the left eye (Fig. 1E&F). The right eye showed no cells in the anterior chamber nor endothelial precipitates or hypopyon. In the left eye, 1 + cells were observed in the anterior chamber without endothelial precipitates or hypopyon. IOP was 8 mmHg in the right eye and 11 mmHg in the left eye. The fundus appeared blurred, but no abnormalities were evident. The patient had no history of contact lens use, trauma, eye surgery, or herpetic eye disease. The right eye was amblyopic. Three months before the onset of ocular symptoms, the patient was started on ruxolitinib 10 mg twice daily, for JAK2 + primary myelofibrosis. Two weeks prior to the onset of ocular symptoms, the ruxolitinib dose was increased to 20 mg twice daily. The patient was not taking any other immunosuppressive medications. The ocular treatment was changed to topical ganciclovir 0.15% ointment five times daily, ofloxacin eye drops three times daily (prophylactically), and PFAT six times daily in both eyes, in addition to oral valacyclovir 2 g per day. Corneal swabs from both eyes for viral RealTime PCR confirmed the presence of HSV-1 (Ct value 24.69 for the right eye & Ct value 25.36 for the left eye) and the absence of VZV. Following consultation with the hematology department, ruxolitinib dosage was reduced to 10 mg daily. The topical and oral antiviral therapy was slowly tapered off. Two weeks after presentation, a clear reduction in the epithelial defect size was observed, along with an increase in endothelial precipitates in both eyes. For this reason, topical dexamethasone 0.1% eye drops were started once daily. One month after presentation, BCVA was 20/200 in the right (amblyopic) eye and 20/25 in the left eye. Due to persistence of residual epithelial defects in both eyes, 1 IU/ml insulin eye drops were started four times a day (Table 1). Unfortunately, the patient died three weeks later.

### Case 4

A 55-year-old man who had suffered from atopic dermatitis since childhood was prescribed abrocitinib 200 mg once daily via the dermatology department. He had previously undergone treatment with dupilumab, but this was discontinued due to dupilumab-associated ocular surface disease. Seven months after commencing abrocitinib treatment, the patient developed a severe herpetic keratitis. This was diagnosed and treated by his own ophthalmologist. A large geographic corneal ulcer with dendritic extensions, raised edges and stromal thinning was diagnosed in the right eye. The epithelial defect affected the entire vertical corneal diameter. After commencing treatment with oral acyclovir at a dose of 800 mg three times daily and topical PFAT, the epithelial defect closed after three weeks. Oral acyclovir 400 mg once daily was continued as prophylaxis. The patient had no history of contact lenses, trauma or ocular surgery. Twenty years earlier, the patient had experienced a single episode of suspected herpetic keratitis in the same eye. The patient was not taking any other immunosuppressive medication. Six months later, the patient was referred by his ophthalmologist due to a recurrence of herpetic keratouveitis in his right eye despite acyclovir prophylaxis. Three weeks prior to referral, his own ophthalmologist had already started topical dexamethasone 0.1% three times a day and increased the acyclovir dose to 800 mg three times a day. Upon presentation at our clinic, slit lamp exam showed ciliary injection, an 8 by 5 mm geographic corneal epithelial defect with whitish stromal infiltration, stromal thinning, and endothelial precipitates inferiorly (Fig. 1G). Visual acuity was limited to hand movements. Corneal sensitivity was normal and symmetric. Ocular treatment was changed to ofloxacin eye drops six times a day, PFAT eight times a day, ganciclovir 0.15% ointment at night and fluorometholone 0.1% eye drops twice a day. Oral valacyclovir at a dose of 3 g per day was started to replace acyclovir, in combination with doxycycline at a dose of 100 mg twice daily. A corneal swab for viral RealTime PCR confirmed the presence of HSV-1 (Ct value 27.64) and the absence of VZV. Following consultation with the dermatology department, abrocitinib was discontinued at that time and replaced with subcutaneous lebrikizumab injections (250 mg) once every 4 weeks. Over the following weeks, the dosage of topical and oral medication was gradually reduced in line with clinical progress. Two months later, the epithelial defect had closed. Four months after referral, progression towards a prepupillary descemetocele was observed, accompanied by pronounced corneal neovascularisation (Fig. 1H). Vision remained limited to counting fingers. Fluorometholone was continued once daily as maintenance treatment, in combination with ganciclovir ointment at night and oral valacyclovir at a dose of 1 gram per day (Table 1).

**Table 1:** Overview of patient characteristics

Characteristic	Case 1	Case 2	Case 3	Case 4
Age (years)	36	54	78	55
Sex	Female	Male	Male	Male
Diagnosis	Atopic dermatitis	Atopic dermatitis	Myelofibrosis	Atopic dermatitis
JAK inhibitor (dose)	Baricitinib 2/4 mg	Upadacitinib 15/30 mg	Ruxolitinib 40 mg	Abrocitinib 200 mg
Time to onset after initiation	4 months	3 years	3 months	7 months
Ocular presentation	Geographic epithelial keratitis (inferior quadrant)	Geographic epithelial keratitis (inferior half)	Geographic epithelial keratitis (vertical, limbus-to-limbus)	Geographic epithelial keratitis (vertical, limbus-to-limbus)
Laterality	Unilateral	Unilateral	Bilateral	Unilateral
PCR results	HSV-1 positive, VZV negative	HSV-1 positive, VZV negative	HSV-1 positive, VZV negative (both eyes)	HSV-1 positive, VZV negative
Culture results	Not performed	*Staphylococcus aureus*	Not performed	Negative
Topical treatment	Ganciclovir 0.15% 5×/day; fluorometholone 0.1% once daily; cyclopentolate 1% twice daily; PFAT hourly	Cefazolin 5% & tobramycin 1.4% hourly; dexamethasone 0.1% once daily; PFAT hourly; AS 20% hourly	Ganciclovir 0.15% 5×/day; ofloxacin 0.3% 3×/day; PFAT 6×/day; dexamethasone 0.1% once daily; insulin 1 IU/mL 4×/day	Ganciclovir 0.15% once daily; ofloxacin 0.3% 6×/day; PFAT 8×/day; fluorometholone 0.1% twice daily
Systemic treatment	Valacyclovir 3 g/day	Valacyclovir 3 g/day	Valacyclovir 2 g/day	Valacyclovir 3 g/day
Long-term valacyclovir treatment	Tapered and discontinued after 6 months	Tapered to 500 mg/day	Not applicable	Tapered to 1000 mg/day
Final BCVA	20/25	20/63	Not available	Counting fingers

Abbreviations: JAK = Janus kinase; HSV-1 = herpes simplex virus type 1; VZV = varicella-zoster virus; PFAT = preservative-free artificial tears; AS = autologous serum; BCVA = best-corrected visual acuity

## Discussion

In this series, we present four cases of severe HSV1-related keratitis in the context of systemic JAKi use. The use of janus kinase inhibitors has been increasing over the past years, with expanded indications. Currently, JAKi are used, amongst other indications, for the treatment of refractory systemic autoimmune diseases [4], myeloproliferative diseases [8] and corticoid-refractory graft-versus-host disease after allogeneic stem cell transplantation [9]. JAKi have also been successfully used to treat autoimmune disease-associated ocular complications such as peripheral ulcerative keratitis, uveitis and scleritis [10, 11]. The JAK-pathway is crucial in response to IL-10, IL-6, IFN-γ and other key cytokines related to the innate or adaptive immunity, which explains why JAK-inhibition increases the risk of infection [12]. The interferon (IFN) system is one of the first lines of defense activated against invading viral pathogens [13]. Type I IFNs, especially IFNα, are produced upon viral detection by pathogen recognition receptors [14]. IFNα activates the JAK/STAT signaling cascade, which results in the production of several interferon-stimulated genes (ISGs). The ISGs work to limit viral replication and establish an overall anti-viral state [13]. IFNγ (type II IFN) is responsible for the long-term maintenance of latency. It is primarily secreted by CD8 + T cells that remain resident in the trigeminal ganglia [15]. IFNγ synergizes with the innate type I IFNs to potently inhibit HSV-1 replication in vitro and in vivo [16]. Animal studies have shown that JAKi decrease the production of antiviral cytokines and chemokines involved in anti–HSV-1 response, such as IFN-α, IFN-γ, and CXCL9/10 [17].

Herpes Zoster reactivation is the most recognised infectious complication of JAKi as a group [4, 18]. A long-term data safety trial in AD with more than 2000 patients treated with baricitinib revealed an incidence rate of 67.2 for general infections, 6.7 for HSV and 2.8 for HZ [19]. Studies have shown a higher incidence of infections for upadacitinib than placebo, especially HZ infection [20]. Ruxolitinib has also been associated with an increased risk of HZ infection in several studies [21]. More recently, Chen et al. [6] investigated the characteristics of abrocitinib-associated herpes virus reactivation through the FDA Adverse Event Reporting System (FAERS). Using the FAERS database, a total of 56 reports were distinctly associated with herpes virus reactivation, of which 4 were classified as ophthalmic herpes simplex without further specification [4]. Results from an analysis of the FAERS database showed that females were at relatively high risk for herpes virus reactivation. Inclusion of dupilumab in combination regimens appeared to reduce the risk of reactivation, while regimens containing baricitinib increased the risk [6]. As suggested by Chen et al. [6], the risk of abrocitinib-induced serious AE related to herpes virus reactivation may have been underestimated due to the rigorous enrollment of clinical populations, the relatively short study duration and inherent susceptibility of AD disease itself. Specifically, herpes simplex events associated to JAKi are reported less frequently than zoster in trial data and have generally received less attention [4].

Interestingly, no studies with detailed descriptions of the morphological characteristics of suspected JAKi-associated herpes simplex keratitis have been published. The above case series appears to support an association between herpes simplex keratitis and the use of oral JAK inhibitors (JAKi). Several arguments support this hypothesis.

Firstly, the four patients presented with similar signs of, PCR-proven, HSV1-related herpetic keratitis. Viral PCR for VZV was negative in all cases. Each patient presented with a geographic epithelial keratitis. Case 3 presented with bilateral involvement. Both the geographic subtype of epithelial herpetic keratitis and the bilateral nature are exceptional within the group of herpes simplex keratitis [22–24]. Geographic epithelial herpetic keratitis is often difficult to treat and requires prolonged therapy [23]. In a retrospective cohort of 544 patients with HSV eye disease, 1.3% presented with bilateral herpetic keratoconjunctivitis, all of whom had concurrent atopy or immune deficiency [22]. Neurotrophic keratopathy is a feared complication of herpetic epithelial keratitis [23]. For the above patients, intensive treatment involving PFAT, autologous serum eye drops and/or insulin eye drops was used to close the corneal epithelium. Ultimately, an amniotic membrane transplant was required in Case 2. Bilateral herpetic keratitis is a rare phenomenon and is known to develop in patients with a compromised immune system [24]. Recently, a case was reported of a patient treated with ruxolitinib for graft-versus-host disease (GVHD) who developed pseudodendritic keratitis related to Herpes Zoster in the right eye and acute retinal necrosis (ARN) in the left eye [25].

Secondly, the patients were treated with various JAKi (baricitinib, upadacitinib, ruxolitinib and abrocitinib), suggesting an underlying class effect. Within this case series, no effect of JAK selectivity could be demonstrated, as both JAK1 (upadacitinib & abrocitinib) and JAK1&2 (baricitinib & ruxolitinib) selective JAKi were associated with HSV1-related keratitis. In upadacitinib-treated patients, studies have shown the rate of Herpes Zoster to be higher with the 30 mg dose (compared to 15 mg) [20]. In Case 3, a similar course was observed, with geographic herpes simplex keratitis developing 2 weeks after the dose of ruxolitinib was increased from 20 mg to 40 mg per day.

Finally, the timing is also suggestive: viral keratitis manifested four months after baricitinib treatment was started in Case 1, two weeks after the ruxolitinib dose was increased in Case 3 (started three months earlier), and seven months after abrocitinib was commenced in Case 4. Only in Case 2, had the patient been using upadacitinib for a longer duration (3 years). It is known that IMIDs, such as atopic dermatitis, are associated with an increased risk of herpes virus reactivation [26]. Sporadic cases of bilateral herpetic keratitis have been described in patients with AD [22]. However, Cases 1, 2 and 4 had been monitored for decades due to severe AD and they only developed geographic HSV1-related keratitis during treatment with JAKi.

The main limitations of the study are the small sample size and the retrospective design. This case series is therefore not intended to prove causality, but rather to highlight the association between oral JAKi use and the development of herpetic keratitis. Increased awareness of this association among ophthalmologists, dermatologists, hematologists, and other prescribing clinicians can lead to more comprehensive patient counseling. Furthermore, awareness among both patients and physicians can facilitate the early recognition of herpetic keratitis symptoms.

Monitoring the risk of infections is advised for patients using JAKi, especially in elderly patients and patients with comorbidities, under prolonged treatment, receiving high doses of JAKi or using high doses of corticosteroids [6, 20]. Patients with AD treated with JAKi should be considered for VZV vaccination [26]. In our Case Series, only Case 1 had received prior VZV vaccination. VZV and HSV-1 share approximately 65 homologous genes, resulting in T-cell cross-reactivity, whereby immune cells can recognize both viruses in a laboratory setting [27]. However, differences in surface proteins prevent cross-protection. VZV vaccines primarily target glycoprotein E, while HSV-1 immunity is predominantly reliant on glycoprotein D, a protein VZV lacks entirely [28]. Studies have demonstrated that VZV vaccination is ineffective at efficiently boosting the specific CD8 + T cells conserved between VZV and HSV-1 [29]. Atopy, immune deficiencies, autoimmune diseases, long-term immunosuppression, corticosteroid use, organ transplantation, emotional stress, fever, ultraviolet radiation exposure and ocular (accidental or surgical) trauma have also been related to reactivation of herpes simplex virus [24]. Physicians prescribing JAKi should screen for these HSV risk factors or a history of HSV and consider oral antiviral prophylaxis for high-risk patients.

## Conclusions

In conclusion, this case series provides further evidence of the need to monitor patients receiving oral JAK inhibitors for signs of herpetic keratitis. In addition to the risk of developing Herpes Zoster infections, clinicians also need to be aware of the increased risk of herpes simplex virus-related herpetic keratitis. Not only can HSV1-related keratitis manifest itself in this patient group as a severe geographic keratitis, it can also occur bilaterally. Treatment typically involves a combination of artificial tears, autologous serum eye drops and/or topical steroids, alongside topical and oral antiviral medication. If patients show a good systemic response to JAKi therapy, but exhibit risk factors for developing herpetic complications, it is recommended that they receive oral antiviral prophylaxis. Based on this case series, we recommend an urgent ophthalmological evaluation for patients taking oral JAKi who present with unilateral or bilateral red, painful eye(s), even in the absence of periocular signs of Herpes Zoster infection. 

## Data Availability

The original contributions presented in this study are included in this article material; further inquiries can be directed to the corresponding author.

## Abbreviations

AD: Atopic Dermatitis
ARN: Acute Retinal Necrosis
BCVA: Best-Corrected Visual Acuity
CI: Confidence Interval
EMA: European Medical Agency
FAERS: FDA Adverse Event Reporting System
GP: General Practitioner
GVHD: Graft-versus-Host Disease
HR: Hazard Ratio
HSV-1: Herpes Simplex Virus type 1
HZ: Herpes Zoster
IFN-γ: Interferon Gamma
IL: Interleukin
IMIDs: Immune Mediated Inflammatory Diseases
IOP: Intraocular Pressure
ISG: Interferon-Stimulated Genes
JAKi: Janus Kinase Inhibitors
PCR: Polymerase Chain Reaction
PFAT: Preservative-free Artificial Tears
RA: Rheumatoid Arthritis
SpA: Spondyloarthritis
UC: Ulcerative Colitis
VZV: Varicella Zoster Virus
